# Supplementary figures and images for: Genome-wide analysis in UK Biobank identifies four loci associated with mood instability and genetic correlation with major depressive disorder, anxiety disorder and schizophrenia
Source: Transl Psychiatry. 2017 Nov 30;7:1264. doi: 10.1038/s41398-017-0012-7 (PMC5802589; doi:10.1038/s41398-017-0012-7)

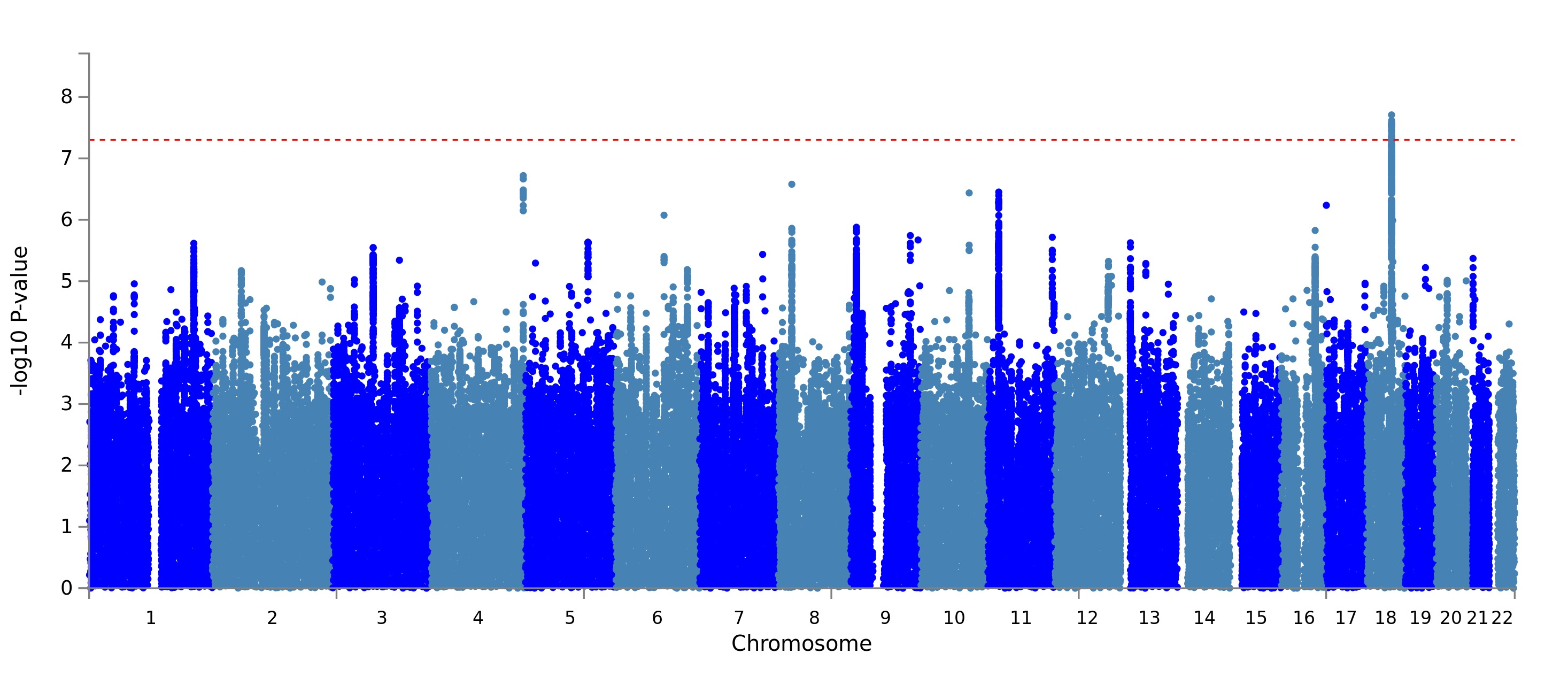

Supplement: Supplementary file 1 — Figure S1 [file 41398_2017_12_MOESM1_ESM.tif]

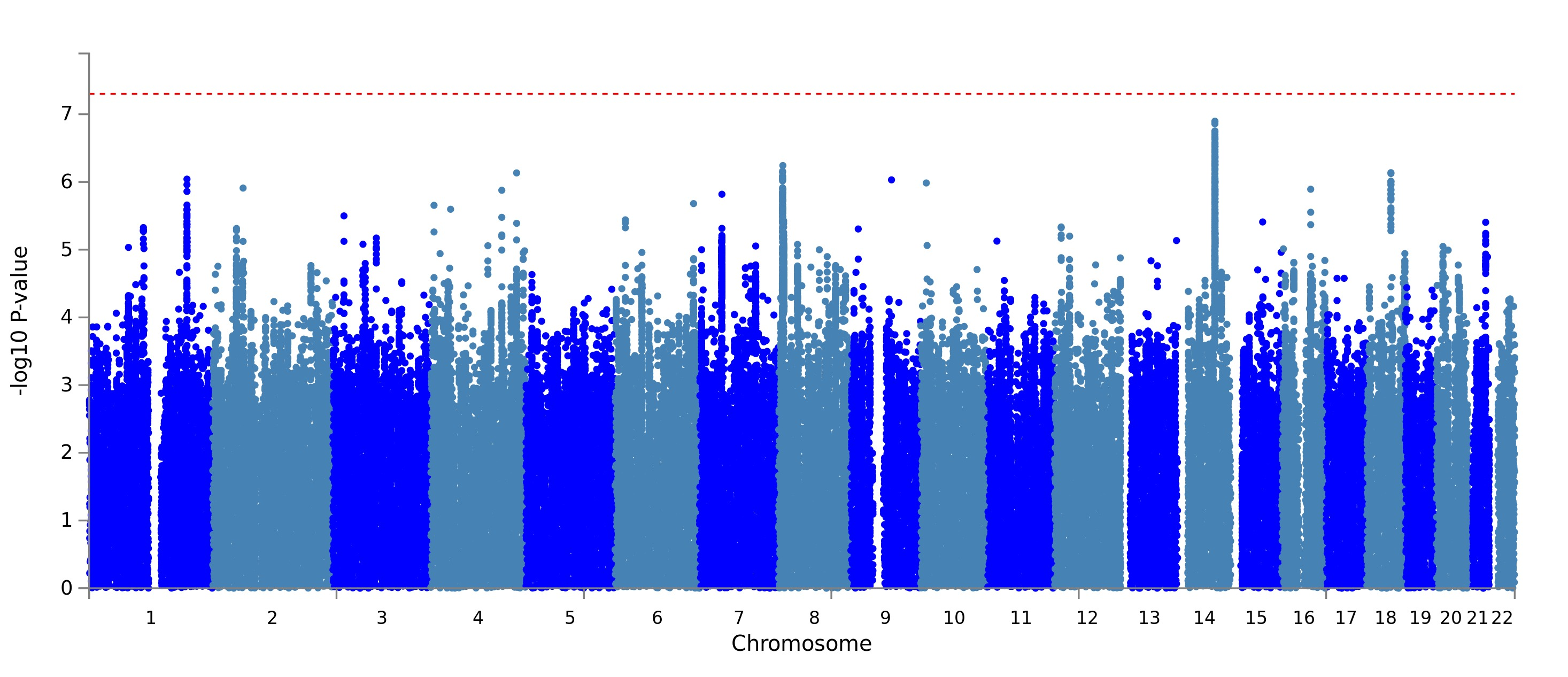

Supplement: Supplementary file 2 — Figure S2 [file 41398_2017_12_MOESM2_ESM.tif]

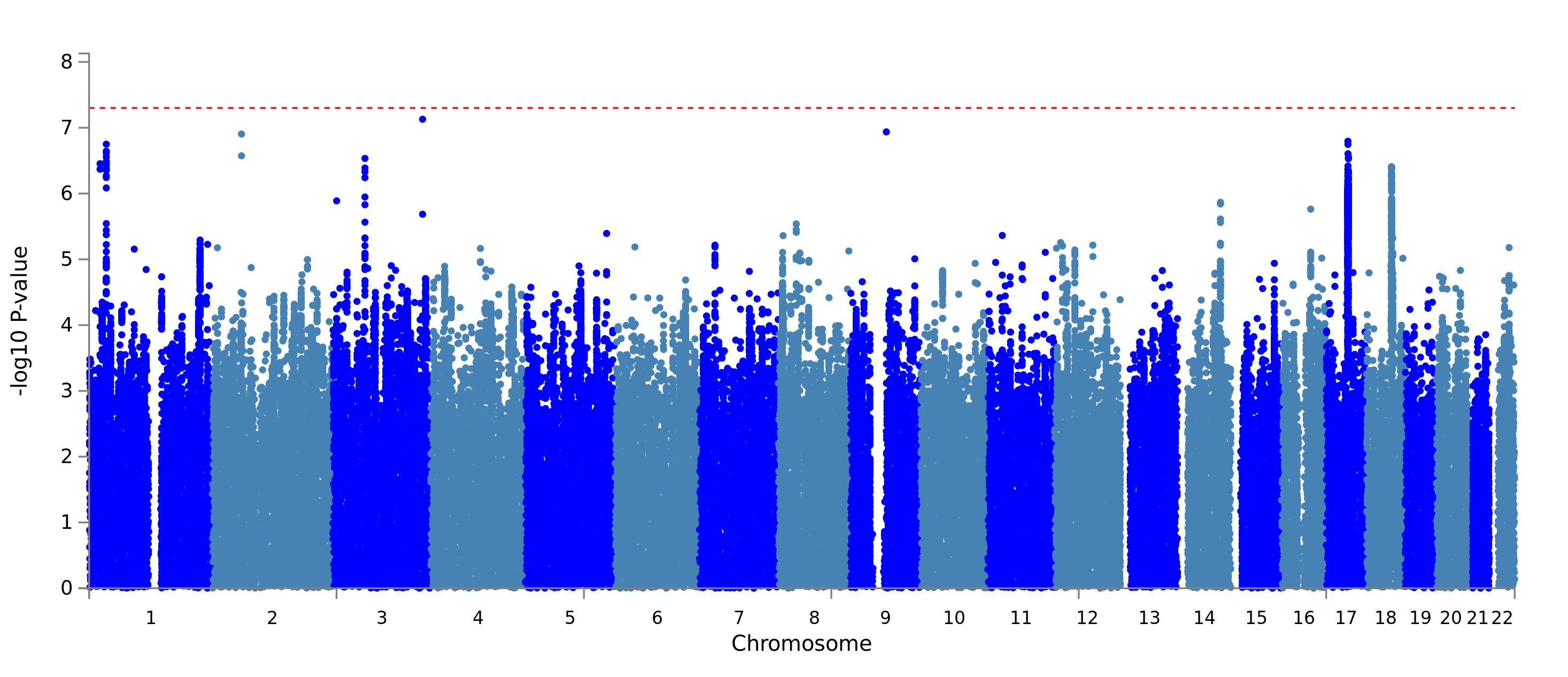

Supplement: Supplementary file 3 — Figure S3 [file 41398_2017_12_MOESM3_ESM.tif]

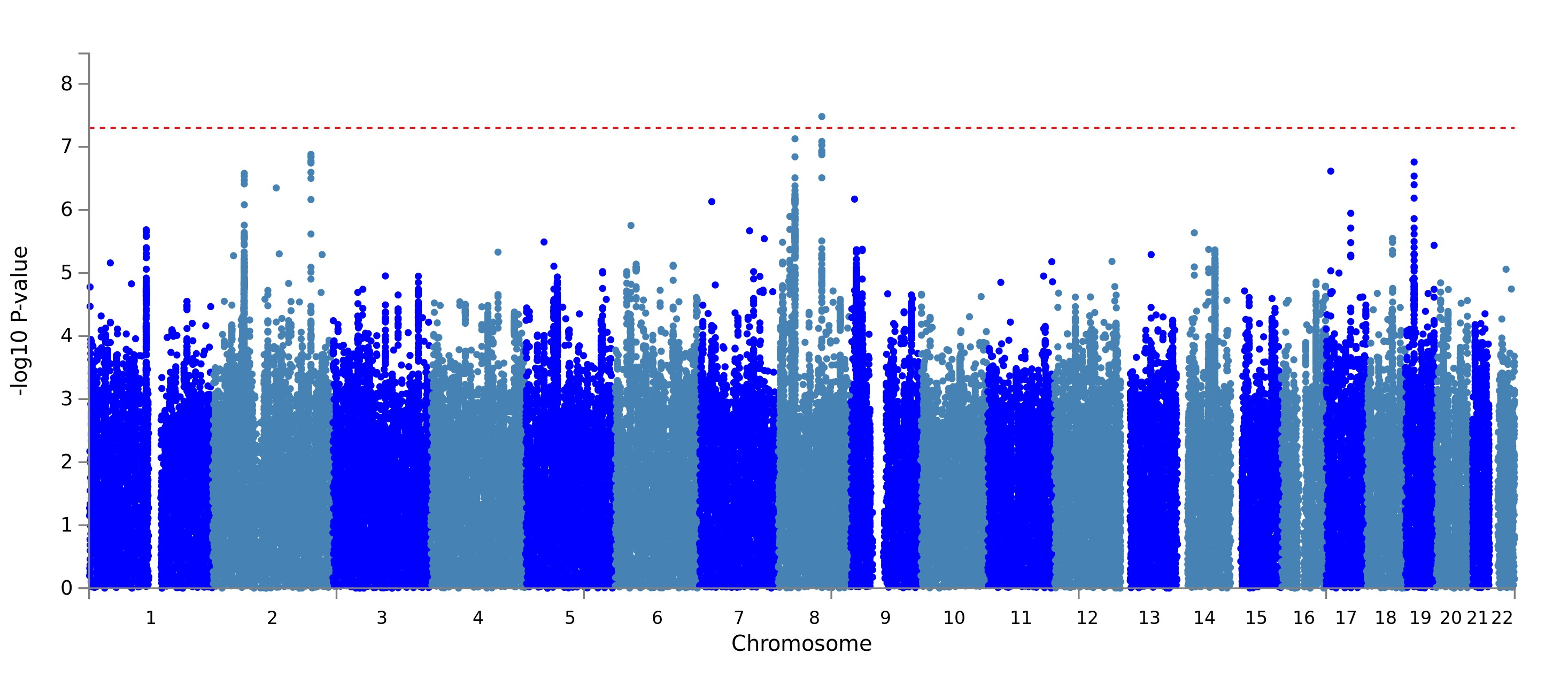

Supplement: Supplementary file 4 — Figure S4 [file 41398_2017_12_MOESM4_ESM.tif]
