# Supplementary material for: Genome-wide analysis in UK Biobank identifies four loci associated with mood instability and genetic correlation with major depressive disorder, anxiety disorder and schizophrenia
Source: Transl Psychiatry. 2017 Nov 30;7:1264. doi: 10.1038/s41398-017-0012-7 (PMC5802589; doi:10.1038/s41398-017-0012-7)
Supplement: Supplementary file 5 — Supplementary Tables S1-S6 [file 41398_2017_12_MOESM5_ESM.docx]

Figure S1. Manhattan plot of GWAS of mood instability in UK Biobank (males only).

Figure S2. Manhattan plot of GWAS of mood instability in UK Biobank (females only).

Figure S3. Manhattan plot of GWAS of mood instability in UK Biobank (age 58 and below).

Figure S4. Manhattan plot of GWAS of mood instability in UK Biobank (age 59 and above).

**Supplementary material**

**Table S1. Genome-wide significant loci associated with mood instability in UK Biobank (excluding 9,865 participants with psychiatric disorder)**

| **Index SNP** | **Chr** | **Position** | **Risk Allele/Other Allele** | **RAF** | **Beta (SE)** | **P-value** |
| --- | --- | --- | --- | --- | --- | --- |
| rs7829975 | 8 | 8,548,117 | A/T | 0.523 | 0.052 (0.009) | 5.32 x 10^-9^ |
| rs10959826 | 9 | 11,459,410 | G/A | 0.789 | 0.055 (0.01) | 4.77 x 10^-7^ |
| rs397852991 | 14 | 75,268,920 | C/CA | 0.673 | 0.045 (0.009) | 1.25 x 10^-6^ |
| rs8084280 | 18 | 50,726,749 | T/A | 0.514 | 0.047 (0.008) | 1.35 x 10^-7^ |

*Shown are LD-independent genome-wide significant SNP associations for mood instability (sorted by genomic position according to NCBI Build 37). Chromosome (Chr) and Position denote the location of the index SNP. RAF = risk allele frequency. Beta = logistic regression coefficient for allele1, SE = standard error for Beta. P-value = the probability of getting the derived test statistic under the null hypothesis. The final column indicates protein-coding genes at the associated loci (see regional plots in supplementary information) or, where there are no genes at the associated locus, the nearest gene if less than 1 MB from the locus.*

**Table S2.** **Psychiatric polygenic risk score analysis of mood instability (adjusted for age, sex, genotyping chip and PGCs 1-8; n_total_=104,103, n_cas_=43,660, n_con_=60,443)**

| **Predictor** | **P** | **Beta** | **SE** | **OR** | **conf lower** | **conf upper** | **Nagelkerke r^2^** |
| --- | --- | --- | --- | --- | --- | --- | --- |
| MDD_0.01 | 1.88*10^-11^ | 0.0148 | 0.00221 | 1.0149 | 1.011 | 1.0193 | 0.0216 |
| MDD_0.05 | 5.47*10^-22^ | 0.0216 | 0.00224 | 1.0218 | 1.02 | 1.0263 | 0.0221 |
| MDD_0.1 | 1.11*10^-26^ | 0.0242 | 0.00226 | 1.0244 | 1.02 | 1.029 | 0.0224 |
| MDD_0.5 | 1.00*10^-34^ | 0.0281 | 0.00229 | 1.0285 | 1.02 | 1.0332 | 0.0229 |
| bipolar_gws | 3.64*10^-01^ | 0.00201 | 0.00221 | 1.002 | 0.99768 | 1.0064 | 0.021 |
| bipolar_0.01 | 1.67*10^-01^ | 0.00305 | 0.00221 | 1.0031 | 0.99873 | 1.0074 | 0.021 |
| bipolar_0.05 | 1.28*10^-01^ | 0.00338 | 0.00222 | 1.0034 | 0.99903 | 1.0078 | 0.021 |
| bipolar_0.1 | 1.65*10^-01^ | 0.00311 | 0.00224 | 1.0031 | 0.99872 | 1.0075 | 0.021 |
| bipolar_0.5 | 1.00*10^-01^ | 0.0037 | 0.00225 | 1.0037 | 0.99929 | 1.0081 | 0.021 |
| SCZ_gws | 1.08*10^-01^ | 0.0035 | 0.00218 | 1.0035 | 0.99924 | 1.0078 | 0.021 |
| SCZ_0.01 | 1.55*10^-03^ | 0.00718 | 0.00227 | 1.0072 | 1.0027 | 1.0117 | 0.0211 |
| SCZ_0.05 | 1.19*10^-04^ | 0.00885 | 0.0023 | 1.0089 | 1.0044 | 1.0134 | 0.0211 |
| SCZ_0.1 | 6.71*10^-05^ | 0.0092 | 0.00231 | 1.0092 | 1.0047 | 1.0138 | 0.0212 |
| SCZ_0.5 | 1.24*10^-04^ | 0.00893 | 0.00233 | 1.009 | 1.0044 | 1.0136 | 0.0212 |

Shown are the results of a logistic regression using psychiatric PRS over a range of P value cut offs split into deciles. Predictor = The PRS used as a predictor in the model in the format “Psychiatric condition _ p value cut off”, P= the P value of the PRS predictor, Beta= the coefficient of the PRS predictor, SE = the standard error of the PRS predictor, OR= the odds ratio of the PRS predictor, conf lower and conf upper = the upper and lower 95% confidence interval of the PRS predictor, Nagelkerke r2 = the variance explained by the whole model.

**Table S3. Psychiatric polygenic risk score analysis of mood instability in females (adjusted for age, genotyping chip and PGCs 1-8; n_total_=53,279, n_cas_=23,308, n_con_=29,971)**

| **Predictor** | **P** | **Beta** | **SE** | **OR** | **conf lower** | **conf upper** | **Nagelkerke R2** |
| --- | --- | --- | --- | --- | --- | --- | --- |
| MDD_0.01 | 8.80*10^-06^ | 0.0137 | 0.00308 | 1.0138 | 1.0077 | 1.0199 | 0.024668 |
| MDD_0.05 | 1.17*10^-10^ | 0.0201 | 0.00312 | 1.0203 | 1.0141 | 1.0266 | 0.025205 |
| MDD_0.1 | 2.96*10^-12^ | 0.022 | 0.00315 | 1.0222 | 1.0159 | 1.0286 | 0.025383 |
| MDD_0.5 | 2.89*10^-17^ | 0.027 | 0.0032 | 1.0274 | 1.021 | 1.0338 | 0.025945 |
| bipolar_gws | 6.20*10^-01^ | 0.00153 | 0.00309 | 1.0015 | 0.99549 | 1.0076 | 0.024186 |
| bipolar_0.01 | 5.35*10^-02^ | 0.00593 | 0.00307 | 1.0059 | 0.99991 | 1.012 | 0.024272 |
| bipolar_0.05 | 1.01*10^-01^ | 0.00509 | 0.0031 | 1.0051 | 0.99901 | 1.0112 | 0.024246 |
| bipolar_0.1 | 1.27*10^-01^ | 0.00475 | 0.00312 | 1.0048 | 0.99864 | 1.0109 | 0.024237 |
| bipolar_0.5 | 6.92*10^-02^ | 0.00569 | 0.00313 | 1.0057 | 0.99955 | 1.0119 | 0.024261 |
| SCZ_gws | 3.43*10^-01^ | 0.00287 | 0.00303 | 1.0029 | 0.99694 | 1.0088 | 0.024202 |
| SCZ_0.01 | 1.62*10^-02^ | 0.00759 | 0.00316 | 1.0076 | 1.0014 | 1.0139 | 0.024323 |
| SCZ_0.05 | 7.32*10^-03^ | 0.00857 | 0.0032 | 1.0086 | 1.0023 | 1.015 | 0.024357 |
| SCZ_0.1 | 5.90*10^-03^ | 0.00885 | 0.00321 | 1.0089 | 1.0026 | 1.0153 | 0.024367 |
| SCZ_0.5 | 1.47*10^-02^ | 0.00791 | 0.00324 | 1.0079 | 1.0016 | 1.0144 | 0.024327 |

Shown are the results of a logistic regression using psychiatric PRS over a range of P value cut offs split into deciles. Predictor = The PRS used as a predictor in the model in the format “Psychiatric condition _ p value cut off”, P= the P value of the PRS predictor, Beta= the coefficient of the PRS predictor, SE = the standard error of the PRS predictor, OR= the odds ratio of the PRS predictor, conf lower and conf upper = the upper and lower 95% confidence interval of the PRS predictor, Nagelkerke r2 = the variance explained by the whole model.

**Table S4. Psychiatric polygenic risk score analysis of mood instability in males only (adjusted for age, genotyping chip and PGCs 1-8, n_total_=50,824, n_cas_=24,804, n_con_=27,939)**

| **Predictor** | **P** | **Beta** | **SE** | **OR** | **conf lower** | **conf upper** | **Nagelkerke R2** |
| --- | --- | --- | --- | --- | --- | --- | --- |
| MDD_0.01 | 4.42*10^-07^ | 0.016 | 0.00317 | 1.0162 | 1.0099 | 1.0225 | 0.01567 |
| MDD_0.05 | 4.99*10^-13^ | 0.023 | 0.00322 | 1.0235 | 1.0171 | 1.03 | 0.016372 |
| MDD_0.1 | 2.69*10^-16^ | 0.027 | 0.00324 | 1.0269 | 1.0204 | 1.0334 | 0.016762 |
| MDD_0.5 | 3.01*10^-19^ | 0.03 | 0.00329 | 1.0299 | 1.0233 | 1.0366 | 0.017115 |
| bipolar_gws | 4.30*10^-01^ | 0.003 | 0.00317 | 1.0025 | 0.99629 | 1.0088 | 0.015015 |
| bipolar_0.01 | 9.96*10^-01^ | -0.00001 | 0.00317 | 0.99999 | 0.99379 | 1.0062 | 0.014999 |
| bipolar_0.05 | 6.48*10^-01^ | 0.001 | 0.00319 | 1.0015 | 0.99521 | 1.0077 | 0.015004 |
| bipolar_0.1 | 6.98*10^-01^ | 0.001 | 0.00321 | 1.0012 | 0.99496 | 1.0076 | 0.015003 |
| bipolar_0.5 | 6.45*10^-01^ | 0.001 | 0.00323 | 1.0015 | 0.99517 | 1.0079 | 0.015004 |
| SCZ_gws | 1.82*10^-01^ | 0.004 | 0.00314 | 1.0042 | 0.99805 | 1.0104 | 0.015046 |
| SCZ_0.01 | 4.17*10^-02^ | 0.007 | 0.00326 | 1.0067 | 1.0003 | 1.0131 | 0.015108 |
| SCZ_0.05 | 6.18*10^-03^ | 0.009 | 0.00331 | 1.0091 | 1.0026 | 1.0157 | 0.015196 |
| SCZ_0.1 | 4.26*10^-03^ | 0.01 | 0.00332 | 1.0095 | 1.003 | 1.0161 | 0.015214 |
| SCZ_0.5 | 2.80*10^-03^ | 0.01 | 0.00335 | 1.0101 | 1.0035 | 1.0167 | 0.015234 |

Shown are the results of a logistic regression using psychiatric PRS over a range of P value cut offs split into deciles. Predictor = The PRS used as a predictor in the model in the format “Psychiatric condition _ p value cut off”, P= the P value of the PRS predictor, Beta= the coefficient of the PRS predictor, SE = the standard error of the PRS predictor, OR= the odds ratio of the PRS predictor, conf lower and conf upper = the upper and lower 95% confidence interval of the PRS predictor, Nagelkerke r2 = the variance explained by the whole model.

**Table S5.** **Psychiatric polygenic risk score analysis of mood instability in those equal to or younger than the median age of 58 (adjusted for age, sex genotyping chip and PGCs 1-8; n_total_=52,743, n_cas_=24,804, n_con_=27,939)**

| **Predictor** | **P** | **Beta** | **SE** | **OR** | **conf lower** | **conf upper** | **Nagelkerke R2** |
| --- | --- | --- | --- | --- | --- | --- | --- |
| MDD_0.01 | 1.80*10^-06^ | 0.0146 | 0.00305 | 1.0147 | 1.0086 | 1.0208 | 0.012342 |
| MDD_0.05 | 9.65*10^-13^ | 0.0221 | 0.0031 | 1.0224 | 1.0162 | 1.0286 | 0.013048 |
| MDD_0.1 | 8.28*10^-16^ | 0.0252 | 0.00313 | 1.0255 | 1.0192 | 1.0318 | 0.013397 |
| MDD_0.5 | 1.13*10^-22^ | 0.0311 | 0.00317 | 1.0316 | 1.0252 | 1.038 | 0.014181 |
| bipolar_gws | 8.77*10^-01^ | 0.000475 | 0.00306 | 1.0005 | 0.99449 | 1.0065 | 0.011771 |
| bipolar_0.01 | 2.51*10^-01^ | 0.00351 | 0.00305 | 1.0035 | 0.99753 | 1.0095 | 0.011803 |
| bipolar_0.05 | 1.35*10^-01^ | 0.00459 | 0.00308 | 1.0046 | 0.99857 | 1.0107 | 0.011826 |
| bipolar_0.1 | 2.66*10^-01^ | 0.00344 | 0.00309 | 1.0034 | 0.99738 | 1.0095 | 0.011801 |
| bipolar_0.5 | 1.44*10^-01^ | 0.00454 | 0.00311 | 1.0046 | 0.99844 | 1.0107 | 0.011824 |
| SCZ_gws | 1.73*10^-02^ | 0.00719 | 0.00302 | 1.0072 | 1.0013 | 1.0132 | 0.011912 |
| SCZ_0.01 | 7.10*10^-03^ | 0.00845 | 0.00314 | 1.0085 | 1.0023 | 1.0147 | 0.011952 |
| SCZ_0.05 | 4.49*10^-04^ | 0.0112 | 0.00318 | 1.0112 | 1.0049 | 1.0176 | 0.012079 |
| SCZ_0.1 | 4.34*10^-04^ | 0.0112 | 0.0032 | 1.0113 | 1.005 | 1.0177 | 0.012081 |
| SCZ_0.5 | 5.86*10^-04^ | 0.0111 | 0.00323 | 1.0112 | 1.0048 | 1.0176 | 0.012067 |

Shown are the results of a logistic regression using psychiatric PRS over a range of P value cut offs split into deciles. Predictor = The PRS used as a predictor in the model in the format “Psychiatric condition _ p value cut off”, P= the P value of the PRS predictor, Beta= the coefficient of the PRS predictor, SE = the standard error of the PRS predictor, OR= the odds ratio of the PRS predictor, conf lower and conf upper = the upper and lower 95% confidence interval of the PRS predictor, Nagelkerke r2 = the variance explained by the whole model.

**Table S6. Psychiatric polygenic risk score analysis of mood instability in those older than the median age of 58 (adjusted for age, sex genotyping chip and PGCs 1-8, n_total_=51,360, n_cas_=18,856, n_con_=23,504)**

| **Predictor** | **P** | **Beta** | **SE** | **OR** | **conf lower** | **conf upper** | **Nagelkerke R2** |
| --- | --- | --- | --- | --- | --- | --- | --- |
| MDD_0.01 | 2.60*10^-06^ | 0.0151 | 0.0032 | 1.0152 | 1.0088 | 1.0216 | 0.0028399 |
| MDD_0.05 | 7.71*10^-11^ | 0.0211 | 0.00325 | 1.0214 | 1.0149 | 1.0279 | 0.0033778 |
| MDD_0.1 | 1.41*10^-12^ | 0.0232 | 0.00327 | 1.0234 | 1.0169 | 1.03 | 0.0035861 |
| MDD_0.5 | 3.58*10^-14^ | 0.0251 | 0.00331 | 1.0254 | 1.0188 | 1.0321 | 0.0037779 |
| bipolar_gws | 2.54*10^-01^ | 0.00365 | 0.0032 | 1.0037 | 0.99738 | 1.01 | 0.0022874 |
| bipolar_0.01 | 4.03*10^-01^ | 0.00267 | 0.0032 | 1.0027 | 0.99641 | 1.009 | 0.0022715 |
| bipolar_0.05 | 4.95*10^-01^ | 0.0022 | 0.00322 | 1.0022 | 0.9959 | 1.0085 | 0.0022653 |
| bipolar_0.1 | 3.70*10^-01^ | 0.00291 | 0.00324 | 1.0029 | 0.99655 | 1.0093 | 0.0022742 |
| bipolar_0.5 | 3.55*10^-01^ | 0.00301 | 0.00326 | 1.003 | 0.99664 | 1.0094 | 0.0022756 |
| SCZ_gws | 9.12*10^-01^ | -0.000347 | 0.00314 | 0.99965 | 0.99351 | 1.0058 | 0.0022532 |
| SCZ_0.01 | 7.72*10^-02^ | 0.00581 | 0.00329 | 1.0058 | 0.99937 | 1.0123 | 0.0023359 |
| SCZ_0.05 | 5.71*10^-02^ | 0.00633 | 0.00333 | 1.0063 | 0.99981 | 1.0129 | 0.0023491 |
| SCZ_0.1 | 3.71*10^-02^ | 0.00696 | 0.00334 | 1.007 | 1.0004 | 1.0136 | 0.0023684 |
| SCZ_0.5 | 4.85E-02 | 0.00664 | 0.00337 | 1.0067 | 1 | 1.0133 | 0.0023563 |

Shown are the results of a logistic regression using psychiatric PRS over a range of P value cut offs split into deciles. Predictor = The PRS used as a predictor in the model in the format “Psychiatric condition _ p value cut off”, P= the P value of the PRS predictor, Beta= the coefficient of the PRS predictor, SE = the standard error of the PRS predictor, OR= the odds ratio of the PRS predictor, conf lower and conf upper = the upper and lower 95% confidence interval of the PRS predictor, Nagelkerke r2 = the variance explained by the whole model.
